# The Shift of Soil Bacterial Community After Afforestation Influence Soil Organic Carbon and Aggregate Stability in Karst Region

**DOI:** 10.3389/fmicb.2022.901126

**Published:** 2022-06-27

**Authors:** Jiacheng Lan, Shasha Wang, Junxian Wang, Xue Qi, Qixia Long, Mingzhi Huang

**Affiliations:** School of Karst Science/State Engineering Technology Institute for Karst Desertification Control, Guizhou Normal University, Guiyang, China

**Keywords:** afforestation, soil bacterial community, soil aggregate, soil organic carbon, karst region

## Abstract

Soil microbes regulate the carbon cycle and affect the formation and stabilization of soil aggregates. However, the interactions between the soil microbial community and soil organic carbon (SOC) fractions, organic carbon (OC) content in aggregates, and soil aggregate stability after afforestation are remain poorly understood. In our study, we investigated SOC fractions in bulk soil, aggregate-associated OC content, soil aggregate stability, and soil bacterial community with high-throughput 16S rRNA sequencing at sites representing natural secondary forest (NF) and managed forest (MF), with cropland (CL) as reference in a degraded karst region of Southwest China. Our results showed that afforestation remarkably increased the SOC fraction and OC content in aggregates, the mean weight diameter (MWD), and the mean geometric diameter (GMD). The most dominant bacterial phyla detected were *Acidobacteriota*, *Actinobacteriota*, *Proteobacteria*, and *Chloroflexi* across all soils. Afforestation remarkably altered the relative abundances of most of the dominant soil bacteria at the phylum, class, and order levels. Interestingly, such changes in the abundance of soil bacteria taxa had significantly effects on SOC fraction, aggregate-associated OC content, MWD, and MGD. The abundance of dominant bacterial taxa such as *Methylomirabilota*, *Latescibacterota*, *Methylomirabilia*, *MB-A2-108*, *norank_Latescibacterota*; *Dehalococcoidia*, *Rokubacteriales*, *Gaiellales*, *Microtrichales*, *norank_c__MB-A2-108*, *norank_c__norank_p__Latescibacterota*, *Rhizobiales*, and *S085* not only remarkably increased but also had significant positive effects on SOC fractions and aggregate-associated OC content after afforestation. Moreover, MWD and MGD were positively correlated with the relative abundance of *Methylomirabilota*, *Methylomirabilia*, *Rokubacteriales*, *Latescibacterota*, and *Rhizobiales*. Results indicated the importance of certain soil bacteria for regulating SOC storage and soil aggregate stability. We concluded that afforestation on cropland could alter the abundance of soil bacteria, and these changes modulate the stability of soil aggregates and SOC fractions.

## Highlights

– Afforestation remarkably affected soil bacterial abundance and composition.
– Changes in SOC fractions and OC content in aggregates were closely correlated with bacterial abundance.
– Remarkable changes in certain bacterial taxa abundance strongly affected soil aggregate stability.

## Introduction

Afforestation on cropland (CL) has been recognized as an important measure to enhance soil aggregate stability and sequestrate soil organic carbon (SOC) in fragile ecosystems (Wei et al., 2012; Qiu et al., 2015; Hu et al., 2018; Hu and Lan, 2019). As a consequence, afforestation is important to mitigate climate change (Deng et al., 2014), regulate ecosystem restoration (Deng and Shangguan, 2017), and prevent land degradation (Lan, 2020) in terrestrial ecosystems. Numerous studies have explored the effects of afforestation on changes in SOC and its fractions (Pang et al., 2019; Lan, 2020; Zhang et al., 2020) as well as their interaction with soil aggregate fractions and stability (Wei et al., 2012; Qiu et al., 2015; Liu et al., 2020a; Xiao et al., 2021). However, the strength and magnitude of the effects of afforestation on SOC fractions, OC content in aggregates, and soil aggregate stability are still conflicting and have considerable uncertainties. These uncertainties are attributed to a diversity of abiotic and biotic factors including climate, edaphic, plant, and microbial factors. Soil microbes are important for the formation and stabilization of soil aggregates (Six et al., 2004; Garcia-Franco et al., 2015) and SOC dynamics (Zhao et al., 2018b; Wang et al., 2019b). Therefore, shifts in microbial community composition and abundance in response to afforestation may have significant effects on the stability of carbon fractions and soil aggregates. In this sense, a clear understanding of soil microbial community, SOC fraction, soil aggregate stability, and their relationships in response to afforestation is urgently needed.

Soil bacteria, as the most abundant and diverse microbial organisms (Meng et al., 2019), are not only the key regulator of the carbon cycle (Zhao et al., 2018b; Lu et al., 2019) but also have important functions in aggregate formation and stabilization (Rashid et al., 2016; Cania et al., 2019). Land-use changes induce differences in the diversity of plant and the quality of organic matter, leading to different responses of soil microbes to SOC dynamics (Deng et al., 2016). However, conflicts exist in whether soil microbial community affects SOC and its fraction dynamics across land-use change. Afforestation can produce fresh carbon input into soil by increasing plant biomass (Lan et al., 2022). Considerable increase in carbon inputs can promote soil microbial growth or alter microbial diversity, thereby increasing the SOC storage (Trivedi et al., 2015; Ren et al., 2018; Zhao et al., 2018b). By contrast, Fontaine et al. (2007) reported that large amounts of plant residue inputs may decrease the efficiency of carbon utilization or soil organic matter decomposition by soil microbes, ultimately resulting in reduction in soil carbon storage. Therefore, changes in SOC fractions may have positive or negative correlations with some bacterial taxa. For instance, afforestation-induced increases in soil bacterial taxa such as *Proteobacteria*, *Chloroflexi*, *Gemmatimonadetes*, *Bacteroidetes*, and *Acidobacteriota* had strong positive effects on SOC fractions, but those in *Actinobacteriota*, *Firmicutes*, and *Verrucomicrobiota* had negative association with SOC fractions (Zhao et al., 2018b). The higher relative abundance of the major phyla *Proteobacteria*, *Bacteroidetes*, and *Cyanobacteria* was favorable for the increases in SOC fractions, while certain taxa such as *Actinobacteriota* and *Chloroflexi* drove negative responses of SOC fractions (Ren et al., 2018). Nevertheless, which bacterial taxa are closely related to SOC fractions remains unclear. However, these relationships are still unknown at a finer level of bacterial classification. Further study is needed to clarify how changes in certain bacterial taxa at different classification levels effectively explain the changes in SOC fractions. According to the conceptual model of Golchin et al. (1994), fresh and labile organic matter increased the availability of organic matter, thereby promoting microorganism growth; this phenomenon causes rapid stimulation of soil microbiota, increases soil microbial activities, and enhances soil aggregation and soil aggregate stability (Guo et al., 2020; Hu et al., 2020). Changes in soil aggregate stability were closely associated with soil bacterial community (Rahman et al., 2017; Zhao et al., 2018a). The rapid growth and activity of soil bacteria influences the production of exudates and hyphae, mostly involving polysaccharides, which in turn act as a binding agent that integrates soil fine particles into macroaggregates (Tisdall and Oades, 1982; Rahman et al., 2017). The distribution patterns of microbes in different aggregate sizes were different due to the heterogeneity of distinct microbial habitats (Trivedi et al., 2017; Lin et al., 2019). A recent study found that the dominant bacterial phyla had significant positive impacts on OC content in aggregates and on the stability of soil aggregates after afforestation. These results suggest that SOC accumulation and stabilization is regulated by interactions between the bacterial community structure and soil aggregate stability. However, microorganisms can directly consume organic binding agents, which hold aggregates together, leading to their decomposition (Li et al., 2018). Therefore, considerable uncertainties still exist in terms of the interactions between soil microbial community and OC content in soil aggregates and of soil aggregate stability after afforestation. A better understanding of these interactions is essential for sustainable forest management (Zhao et al., 2018a).

The karst landscape in Southwest China covers approximately 0.51 million km^2^ and is known for its highly vulnerable and sensitive ecosystem that suffers from poor agricultural land use and management, which causes soil erosion and rocky desertification (Jiang et al., 2014). Thus, the Grain for Green Project and Karst Rocky Desertification Restoration Project has been implemented by the Chinese government to recover degraded agricultural land and control soil erosion and rocky desertification (Tong et al., 2018). Afforestation is an effective strategy for SOC stocks in the karst region. Numerous studies have focused on the effects of land-use change/vegetation restoration/afforestation on aggregate formation and SOC sequestration (Hu and Lan, 2019; Lan, 2020; Liu et al., 2020a; Xiao et al., 2021; Lan et al., 2022), soil aggregate stability (Lan, 2021), and microbial community (Xue et al., 2017; Qiu et al., 2019; Zhao et al., 2019; Wang et al., 2021; Li et al., 2021b). However, these studies have not investigated the changes in SOC fractions, OC content in aggregates, and soil aggregate stability that are associated with soil bacterial communities. Therefore, in our study, we investigated SOC and its fractions in bulk soil, aggregate-associated OC content, soil aggregate stability, and soil bacterial community at sites representing natural secondary forests (NF) and managed forest (MF, *Zanthoxylum bungeanum* plantations) after afforestation on former cropland (CL) in a karst region in Southwest China. In this study, we hypothesized that afforestation markedly alter the composition and abundance soil bacteria, and the shift in specific bacterial taxa would account for changes in SOC fractions and aggregate stability. The objectives of this study were to (1) determine remarkable changes in bacterial community at different soil bacterial classification levels after afforestation and (2) identify the linkages between such remarkable changes in bacterial taxa and variations in SOC fractions, aggregate-associated OC, and soil aggregate stability following afforestation on CL.

## Materials and Methods

### Site Description

**Figure 1:**
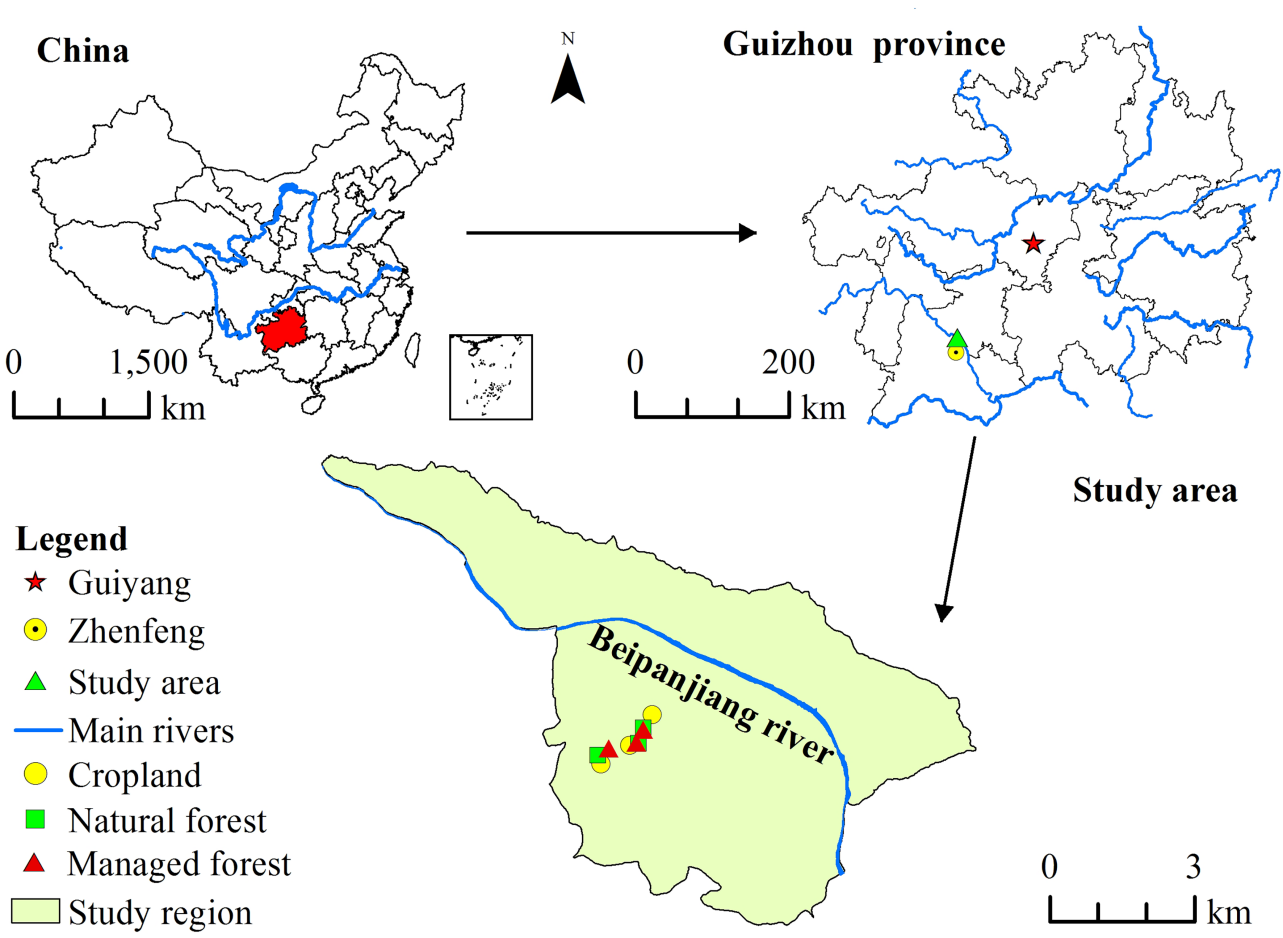
Sketch map location of study area and sampling sites.

This study was conducted in the Huajiang karst ecosystem of Zhenfeng county in Guizhou Province, Southwest China (25°37′20″–25°40′45″ N, 105°37′24″–105°41′30′′ E; Figure 1). The climate is subtropical dry and hot, with an average annual temperature of 18.4°C and a mean annual rainfall of 1,100 mm. Soils in this area are classified as calcareous lime soil (Cheng et al., 2015). The majority of croplands have been gradually converted to natural secondary forest (NF) and managed forest (MF) under the support of the “Grain for Green” project in the late 1990s.

### Experimental Design and Soil Sampling

This experimental site consisted of two afforestation types, namely, natural secondary forest (NF) and managed forest (MF), with cropland as the reference. The croplands had been planted with maize (*Zea mays* L.) for at least 50 years. Fertilizers and animal excreta were applied in March and May irregularly in maize fields each year. Maize was sown in March and harvested in August once a year. The aboveground biomasses of maize were removed from the ground each year. The NF sampling sites were cropland abandoned ~15–20 years and dominated by *Koelreuteria paniculata*, *Toona sinensis*, *Mallotus philippensis*, *Cipadessa baccifera* (Roth.) Miq, etc. The vegetation was restored with the help of natural forces without man-made and reasonable management. Therefore the present vegetation was naturally restored forest. *Z. bungeanum* is the most widely distributed MF and has been planted for about 15–20 years after the cropland was abandoned in our sampling sites. The *Z. bungeanum* plantation was established by moving seedlings about 80-cm long into the field at density of 1,000 trees ha^−1^. The organic and compound fertilizers were usually applied 10 to 30 cm away from the trunk. The fertilization was carried out based on stand age, crown and phenology. The fertilization amount was determined by the tree body and growth stage. The average height is 3 m, and the average crown is 3.5 m × 3 m. Farmers harvest pepper and prune the trees after harvesting from July to September every year. The herbs in the understory of the *Z. bungeanum* plantations are regularly removed.

In November 2018, three sites containing NF, MF, and CL were chosen. The three sites were approximately 0.5–1.5 km apart. Prior to afforestation, all sites were essentially CLs. The NF and MF were converted from adjacent CLs. The mean area of NF, MF and CL sites was ~6,000, 4,000, and 2,500 m^2^, respectively. Within each of the sites, soil types, slopes, gradients, and altitudes in NF, MF, and CL were similar. Three plots in each land-use type were randomly selected. The distance between any two plots exceed 100 m. Soil samples were obtained at 0–10-cm soil depth from five random points following an elongated “S” pattern in each plot and then combined as a homogenized sample. Overall, 27 samples (three land-use types × three sites × three plots) were collected. Each soil sample was immediately transported to the laboratory and divided into three portions, one for aggregate separation and determination of OC in aggregates, one for SOC fraction analysis, and the other one for molecular analysis (stored at −80°C before analysis).

### Analysis of SOC Fractions

A portion of each soil sample was air-dried through 0.25-mm sieves to measure the content of SOC and its fractions in bulk soil. SOC content was analyzed using an elemental analyzer-stable isotope mass spectrometer (Vario ISOTOPE Cube-Isoprime, Elementar, Germany). Particulate organic carbon (POC) was separated based on the modified procedure by Cambardella and Elliott (1992). Easily oxidizable organic carbon (EOC) was measured by a method previously described by Blair et al. (1995). The separation procedures and analysis of POC and EOC were described in detail in our previous study (Lan, 2020). Briefly, 20-g air-dried soil samples were dispersed in 60 ml of 5 g L^−1^ sodium hexametaphosphate by shaking for 18 h. The suspension was wet-sieved through a 53 μm sieve. The material retained on the sieve represents the POC. The POC was dried at 60°C for 24 h in the oven. POC concentrations were measured through oxidation with KCr_2_O_7_ + H_2_SO_4_ and titration with FeSO_4_. The EOC was measured by 333 mmol L^−1^ KMnO_4_. Briefly, air-dried soil samples (containing 15–30 mg of C) and 25 ml of 333 mmol L^−1^ KMnO_4_ were added to a 100 ml tube, and then it was shaken at 250 r min^−1^ for 1 h. After that it was centrifuged at 4,000 r min^−1^ for 5 min to obtain the supernatant. The supernatant was diluted by 1:250 deionized water and measured spectrophotometrically at 565 nm.

### Analysis of Aggregate Fractionation and Aggregate-Associated OC

A portion of each soil sample was separated by wet sieving through 2, 0.25, and 0.053 mm sieves according to Cambardella and Elliott (1993). After separation, soil that retained on 2, 0.25, and 0.053 mm sieves as well as soil that passed through 0.053 mm sieve represent LMA (>2 mm), SMA (0.25–2 mm), MI (0.053–0.25 mm), and <0.053 mm fractions, respectively. The soil in each aggregate fraction was dried at 60°C, weighed to determine the proportion of soil, and ground for analysis of OC content by using an elemental analyzer-stable isotope mass spectrometer (Vario ISOPOTE Cube-Isoprime, Elementar, Germany). The mean recovery of total aggregates remained 96%.

### Biomolecular Analysis

DNA was extracted from 0.15–0.35 g of fresh soil in triplicate with a FastDNA^®^ Spin Kit for Soil (MP Biomedicals, United States). The concentration and quality of DNA were detected using a NanoDrop2000 spectrometer (Thermo Fisher Scientific, USA). The primers 338F (5′-ACTCCTACGGGAGGCAGCAG-3′) and 806R (5′-GGACTACHVGGGTWTCTAAT-3′) targeting the V3–V4 region of the bacterial 16S rRNA gene was used for polymerase chain reaction (PCR). All PCR reactions were performed in triplicate by using a 20 μl reaction mixture containing 1 μl of template DNA, 0.8 μl of each primer (5 μM), 10 μl of 2X Taq Plus Master Mix (Nanjing Nuovizan Biotechnology Co., Ltd.), and 7.4 μl of ddH_2_O. Amplification was conducted under the following thermocycling conditions: 5 min at 95°C for initial denaturation, at 95°C for 30 s, 30 cycles of 30 s primer annealing at 58°C, and elongation at 72°C for 1 min. The amplicons were recovered from 2% agarose gels, purified using AxyPrep DNA Gel Extraction Kit (Axygen Biosciences, Union City, CA, United States), and detected by 2% agarose gel electrophoresis to improve the quality and concentration of the PCR product. The final PCR products were quantified using Quantus^™^ Fluorometer (Promega, United States), and the samples were mixed in an equal amount and adjusted as needed for sequencing. Sequencing libraries were generated using the NEXTFLEX Rapid DNA-Seq Kit. Sequencing was performed on Illumina MiseqPE300 platform at the Major Biological Institute in Shanghai, China. The raw fastq files of the bacterial reads resulting from MiSeq sequencing were filtered by Trimmomatic and merged by FLASH. Operational taxonomic units (OTUs) with a minimum of 97% similarity were clustered using UPARSE version 7.0. All chimeras were eliminated using UCHIME software prior to further analysis. Each sequence was annotated by RDP classifier^1^ according to the SILVA database (version 138/16S-bacteria database) with a confidence threshold of 70%.

### Data and Statistical Analysis

Soil aggregate stability was determined using mean weight diameter (MWD) and mean geometric diameter (MGD). MWD and MGD were calculated according to the method of Kemper and Rosenau (1986):


(1) $$MWD=\overset{n}{\underset{i=1}{\sum }}x_{i}\times w_{i}\;$$

(2) $$MGD=\exp\;\left[\frac{\sum_{i=1}^{n}w_{i}\times lnx_{i}}{\sum_{i=1}^{n}w_{i}}\right]$$

where $w_{i}$ is the proportion (%) of each aggregate fraction, and $x_{i}$ is the average diameter of each aggregate fraction (mm).

SPSS 17.0 statistical software was used for one-way ANOVA and least significant difference (LSD) multiple comparison test (*p* < 0.05) to assess the significance of the effects of afforestation on MWD, MGD, SOC fraction content in bulk soil, and OC content in aggregates. The normality and homogeneity of variances were checked using Shapiro–Wilk and Levene’s test. Kruskal–Wallis *H*-test (*p* < 0.05) was used to analyze significant differences in bacterial abundance in multiple groups of samples. Spearman’s rank correlation analysis was applied to determine the relationship of bacterial abundance with MWD, MGD, SOC fraction content in bulk soil, and OC content in aggregates. Kruskal–Wallis *H*-test and Spearman’s rank correlation analysis were conducted using R v.3.1.3 program.

## Results

### Changes in Soil Aggregate Stability and SOC Fractions

**Figure 2:**
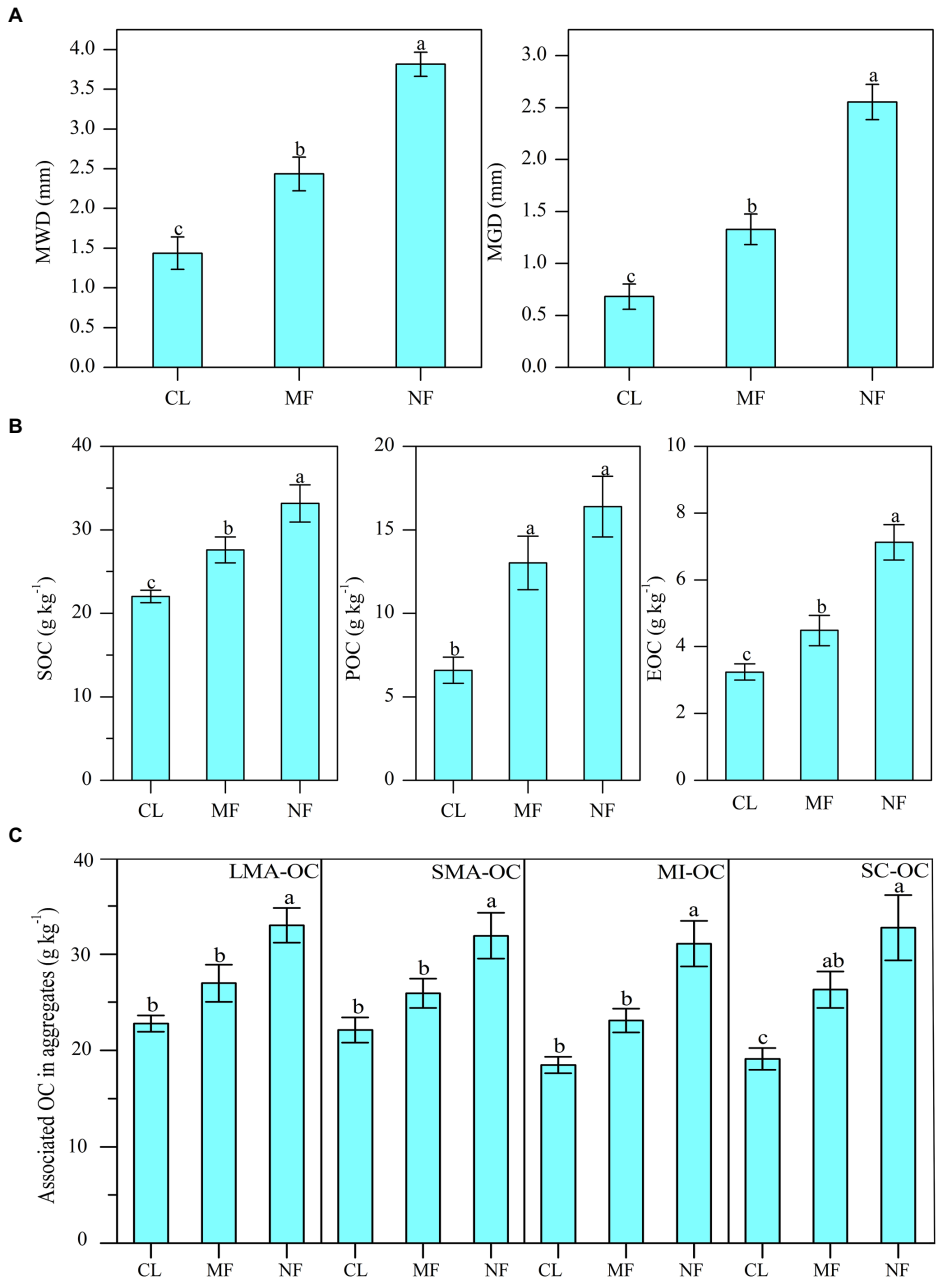
**(A)** Changes of soil aggregate stability index (MWD, GMD); **(B)** soil organic carbon (SOC) and its fractions (POC, EOC) contents in bulk soil and **(C)** the OC contents in aggregates after afforestation. Numerical values are means ± standard errors. Bars with different letters indicate significant differences after afforestation (*p* < 0.05). CL, cropland; MF, managed forest; NF, natural forest; LMA–OC, large macroaggregates associated organic carbon; SMA–OC, small macroaggregates associated organic carbon; MI–OC, microaggregates associated organic carbon; SC–OC, silt + clay organic carbon; POC, particulate organic carbon; EOC, Easily oxidizable organic carbon; MWD, mean weight diameter (mm); MGD, mean geometric diameter (mm).

The MWD and MGD values were significantly increased by 69.5% and 94.9% under MF and by 165.5% and 275% under NF, respectively, compared with those in CL (Figure 2A; *p* < 0.01). The values of MWD and MGD under NF were higher than those under MF by 56.6% and 92.4%, respectively (Figure 2A). Afforestation significantly affected SOC, POC, and EOC in bulk soil, with the highest values under NF, followed by MF and then CL (Figure 2B). The SOC, POC, and EOC contents were increased significantly by 25.9%, 97.6%, and 38.4% under MF and by 50.6%, 148.7%, and 119.9% under NF, respectively, compared with those in CL. We also observed a significant increase in aggregate-associated OC content after afforestation (Figure 2C). The NF sites had 44.7% (*p* < 0.01) LMA-OC, 44.2% (*p* < 0.01) SMA-OC, 68.1% (*p* < 0.01) MI-OC, and 77.1% (*p* < 0.01) SC-OC than the CL sites. The MF sites had 37.5% (*p* < 0.05) SC-OC than the CL sites, whereas no significant differences in LMA-OC, SMA-OC, and MI-OC were observed between the MF and CL sites (Figure 2C).

### Soil Bacterial Community Structure and Compositions

**Figure 3:**
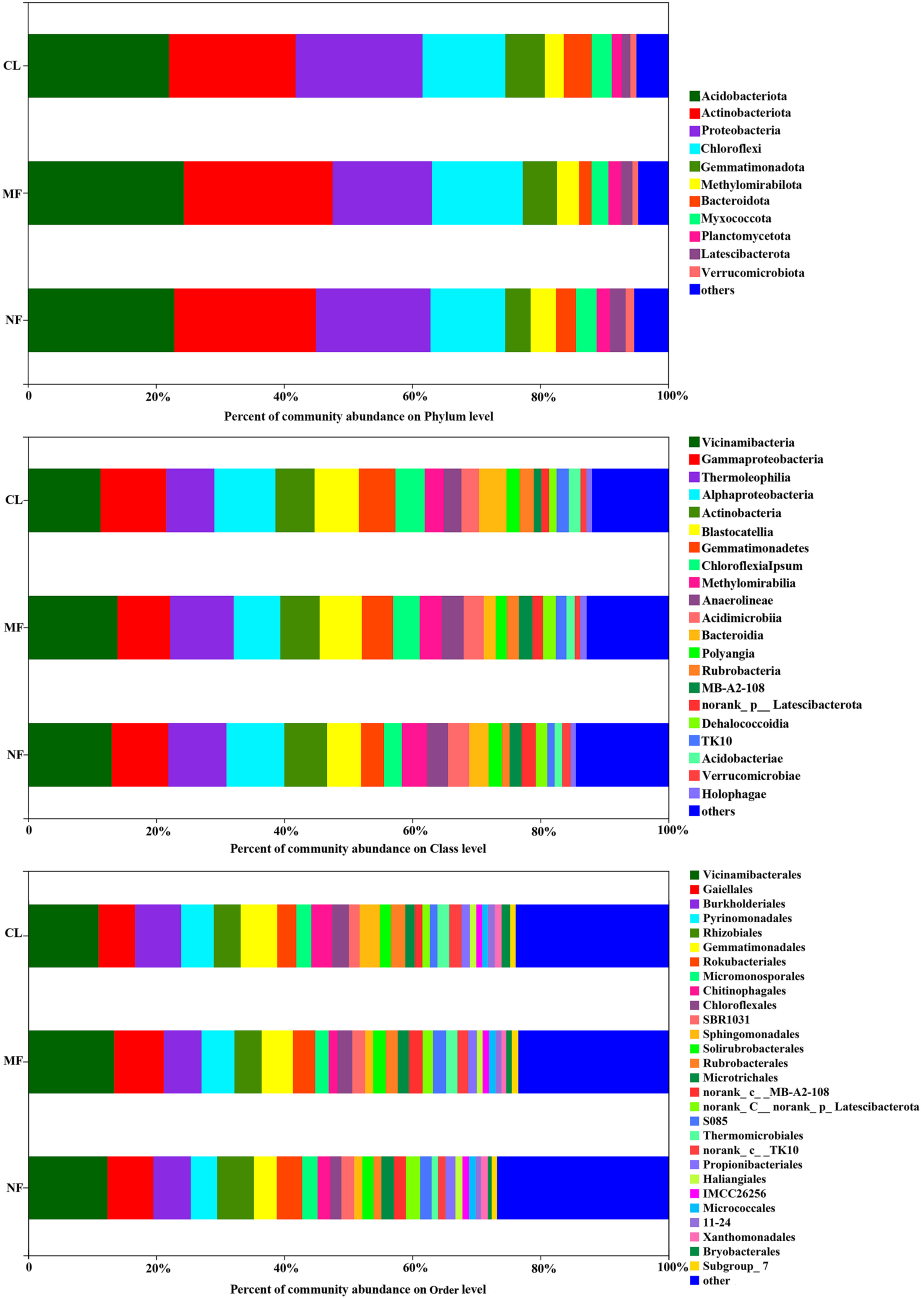
Soil bacterial community composition at phylum, class and order level following afforestation. CL, cropland; MF, managed forest; NF, natural forest.

The dominant soil bacterial phyla (with relative abundance >1%) were *Acidobacteriota* (22.06%–24.39%), *Actinobacteriota* (19.77%–23.22%), *Proteobacteria* (15.50%–19.82%), *Chloroflexi* (11.68%–19.82%), *Gemmatimonadetes* (3.96%–6.15%), *Methylomirabilota* (2.98%–3.96%), *Bacteroidota* (1.94%–4.34%), *Myxococcota* (2.64%–3.25%), *Planctomycetota* (1.61%–2.07%), *Latescibacterota* (1.29%–2.46%), and *Verrucomicrobiota* (0.87%–1.32%) across all soils, accounting for 95.04, 95.31 and 94.69% in CL, MF, and NF, respectively (Figure 3). At the class level, 21 classes were recorded with relative abundance of over 1% in all three land-use types (Figure 3). *Vicinamibacteria* (12.78%), *Gammaproteobacteria* (9.11%), *Thermoleophilia* (8.84%), *Alphaproteobacteria* (8.61%), *Actinobacteria* (6.31%), and *Blastocatellia* (6.29%) were the most dominant class with a relative abundance of >5% in all soils.

**Figure 4:**
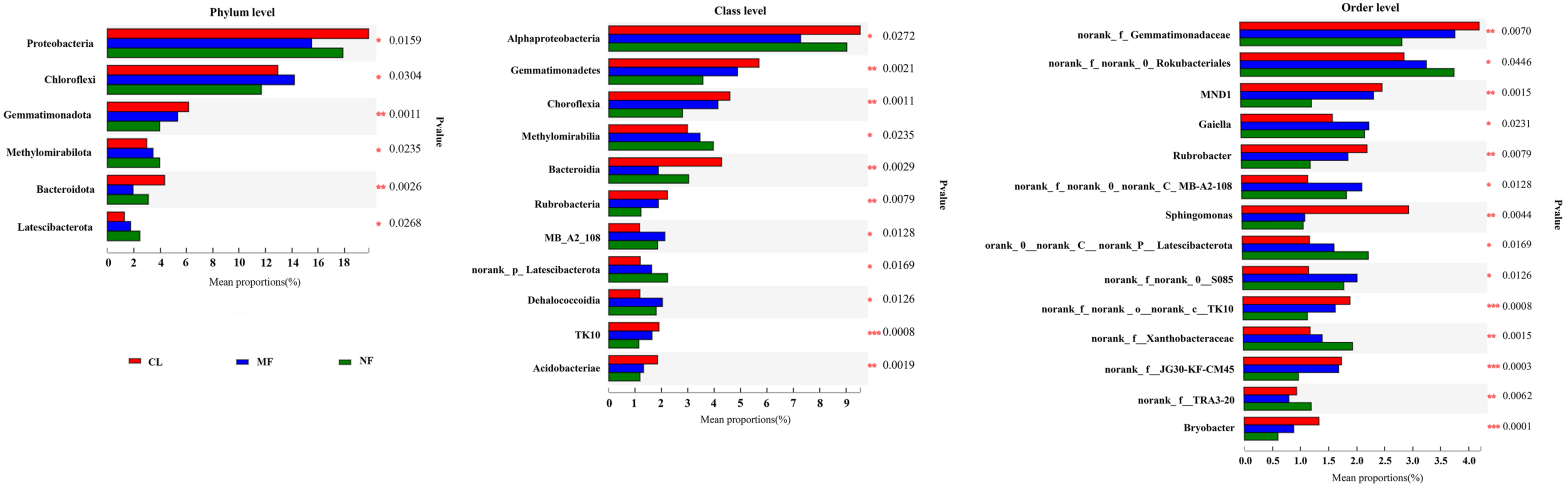
Significant differences of soil bacterial abundance at phylum level, class level and order level after afforestation. CL, cropland; MF, managed forest; NF, natural forest. ^*^p < 0.05; ^**^p < 0.01; ^***^p < 0.001.

The predominant species in the bacterial communities were consistent among the CL, MF, and NF sites. However, the relative abundance of soil bacteria differed after afforestation. At the phylum level, the abundance of *Proteobacteria*, *Chloroflexi*, *Gemmatimonadota*, *Methylomirabilota*, *Bacteroidota*, and *Latescibacterota* changed significantly (*p* < 0.05) after afforestation (Figure 4). The relative abundance of *Proteobacteria*, *Gemmatimonadota,* and *Bacteroidota* was higher in the CL sites than in the afforested sites, whereas *Methylomirabilota* and *Latescibacterota* were relatively more abundant in afforested soil than in the CL sites. Notably, *Actinobacteriota* showed increased trends after afforestation (Figure 3). Greater differences in the composition of the bacterial community were observed at the class level. Among the 21 top classes, 11 dominant classes were observed to have obvious difference after afforestation (Figure 4). The relative abundance of *Methylomirabilia*, *MB-A2-108*, *norank_Latescibacterota*, and *Dehalococcoidia* increased significantly after afforestation, but that of *Alphaproteobacteria*, *Gemmatimonadetes*, *Chloroflexia*, *Bacteroidia*, *Rubrobacteria*, *TK10* and *Acidobacteriae* decreased significantly. Compared with that at the class level, the bacterial community composition exhibited greater differences at the order level. A total of 28 orders were recorded with relative abundance more than 1%, but only 14 orders changed remarkably after afforestation (Figure 4). The abundance of seven orders including *Rokubacteriales* (within *Methylomirabilota* phylum); *Gaiellales*, *Microtrichales*, and *norank_c__MB-A2-108* (within *Actinobacteriota* phylum); *Rhizobiales* (within *Proteobacteria* phylum); *norank_c__norank_p__Latescibacterota* (within *Latescibacterota* phylum); and *S085* (within *Chloroflexi* phylum) was increased significantly by afforestation. Meanwhile, the abundance significantly decreased after afforestation in seven orders specifically *Gemmatimonadaceae* (within *Gemmatimonadota* phylum); *Chitinophagale*s (within *Bacteroidota* phylum); *Sphingomonadales* (within *Proteobacteria* phylum); *Rubrobacterales* (within *Actinobacteriota* phylum); *norank_c__TK10* and *Thermomicrobiales* (within *Chloroflexi* phylum); and *Bryobacterales* (within *Acidobacteriota* phylum).

### Relationships Among OC, Soil Aggregate Stability, and Soil Bacterial Communities

**Figure 5:**
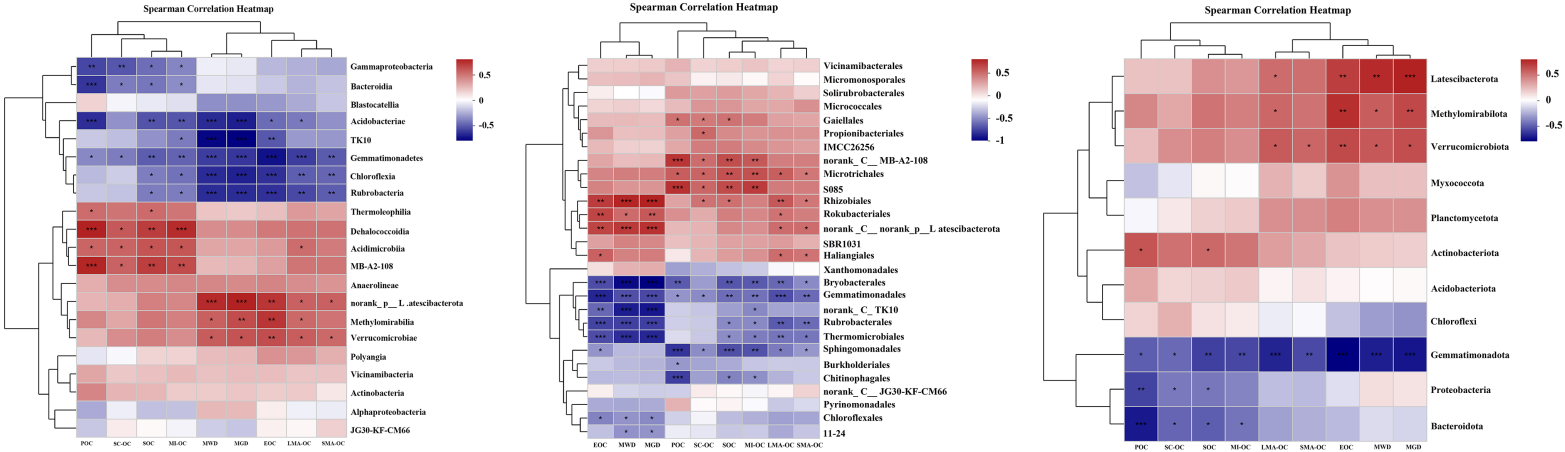
Spearmen correlation heatmap cluster of soil aggregate stability index (MWD, GMD), SOC fractions to the dominant bacterial phylum, class and order after afforestation. LMA–OC, large macroaggregates associated organic carbon; SMA–OC, small macroaggregates associated organic carbon; MI–OC, microaggregates associated organic carbon; SC–OC, silt + clay organic carbon; POC, particulate organic carbon; EOC, Easily oxidizable organic carbon; MWD, mean weight diameter (mm); MGD, mean geometric diameter (mm). ^*^p < 0.05; ^**^p < 0.01; ^***^p < 0.001.

Spearman’s rank correlation coefficients revealed that the changes in SOC fractions, OC in aggregates, and aggregate stability were significantly correlated with soil bacterial communities (Figure 5, *p* < 0.05). At the phylum level, SOC fractions (SOC, POC, and EOC) showed significant positive correlation with the abundance of *Actinobacteriota*, *Methylomirabilota*, *Latescibacterota,* and *Verrucomicrobiota* and negative correlation with the abundance of *Proteobacteria*, *Gemmatimonadota*, and *Bacteroidota*. The relative abundance of *Proteobacteria*, *Gemmatimonadota,* and *Bacteroidota* also had significant negative impacts on OC content in aggregates. LMA-OC and SMA-OC were positively correlated with the abundance of *Methylomirabilota*, *Latescibacterota*, and *Verrucomicrobiota*, but no correlation was found between these bacterial phyla and MI-OC and SC-OC. The abundance of *Methylomirabilota*, *Latescibacterota*, and *Verrucomicrobiota* also exhibited significant positive effects on MWD and MGD. By contrast, *Gemmatimonadota* had a significant negative effect on MWD and MGD.

At the class level, SOC fractions (SOC, POC, and EOC) and aggregate-associated OC were significantly positively correlated with the abundance of *Thermoleophilia*, *Methylomirabilia*, *Acidimicrobiia*, *MB-A2-108*, *norank_p__Latescibacterota*, *Dehalococcoidia*, and *Verrucomicrobiae* in bacterial compositions and were negatively correlated with the abundance of *Gammaproteobacteria*, *Gemmatimonadetes*, *Chloroflexia*, *Bacteroidia*, *Rubrobacteria*, *TK10*, and *Acidobacteriae*. The abundance of *Methylomirabilia*, *norank_ p__Latescibacterota* and *Verrucomicrobiae* also exhibited significant positive effects on MWD and MGD. Meanwhile, *Gemmatimonadetes*, *Chloroflexia*, *Rubrobacteria*, *TK10*, and *Acidobacteriae* had a significant negative effect on MWD and MGD.

At the order level, SOC fractions, aggregate-associated OC, MWD, and MGD showed significant positive correlations with *Gaiellales*, *Propionibacteriales*, *norank_c__MB-A2-108*, *Microtrichales*, *S085*, *Rhizobiales*, *Rokubacteriales*, *norank_c__norank_p__Latescibacterota*, and *Haliangiales*, while were significantly negatively correlated with *Bryobacterales*, *Gemmatimonadales*, *norank_c__TK10*, *Rubrobacterales*, *Thermomicrobiales*, *Sphingomonadales*, *Chitinophagales*, *Chloroflexales* and *11–24*.

## Discussion

### Distinct Change in Soil Bacterial Community After Afforestation

After conversion of CL into forests, the predominant bacteria phyla and classes were the same (Figure 2). The most dominant phylum was Acidobacteria, followed by Actinobacteriota, Proteobacteria, and Chloroflexi in all soils, which were in agreement with previous studies on the conversion of CL into forests (Zhao et al., 2018b; Meng et al., 2019; Wang et al., 2019b, 2021; Zheng et al., 2020; Li et al., 2021b). These similarities in main phyla indicated that afforestation of the former CL in the studied areas may not be sufficient to significantly affect the soil dominant bacterial composition. Similarly, Meng et al. (2019) reported that soil bacterial compositions were very close following forest conversion because all the forest types shared the same history-native broadleaved forest before conversion. Land-use history has a lasting effect on soil bacterial community composition (Guo et al., 2016). However, previous results in karst areas in southwest China found that Verrucomicrobiota (Qiu et al., 2019) and Bacteroidota (Li et al., 2021b) were the most abundant bacteria in soils. The high spatial heterogeneity harbors diverse soil microorganisms (Yuan, 1997; Jiang et al., 2014). The different responses to afforestation were due to the differences in site regions, soil types, land use, afforestation trees, and stages (Kang et al., 2018; Wang et al., 2019b). Moreover, the similar relative abundance of these most dominant bacterial phyla in CL and afforested soil may be due to the fact that these phyla can well adapt to various environments.

Afforestation did not change the dominant phyla and class in the soil bacterial communities, but their abundance was remarkably altered (Figure 3). The differences between CL and the afforestation types were mainly due to *Proteobacteria*, *Chloroflexi*, *Gemmatimonadota*, *Methylomirabilota*, *Bacteroidota*, and *Latescibacterota* at the phylum level, indicating that these phyla were sensitive to environmental changes. The relative abundance of *Proteobacteria*, *Gemmatimonadota*, and *Bacteroidota* was higher in the CL sites than in the afforested sites, while that of *Methylomirabilota* and *Latescibacterota* exhibited an opposite trend (Figure 3). The *Proteobacteria* phylum is usually defined as copiotrophic taxa and can rapidly grow in soils with sufficient labile organic substrates and rich nutrients (Fierer et al., 2007). This finding was confirmed by some studies that the relative abundance of *Proteobacteria* increased after afforestation (Ren et al., 2018; Zhao et al., 2018a; Zheng et al., 2020) and was higher in forest soils than that in non-forest soils (Tripathi et al., 2016; Qiu et al., 2019). However, our finding is in contrast with previous works. The greater relative abundance of *Proteobacteria* but lower SOC in CL indicated that other factors affected *Proteobacteria*. This finding may be explained by the fact that *Proteobacteria* is recognized as one of the largest divisions within prokaryotes (Gupta, 2000) as well as within facultative trophic and aerobic heterotrophic bacteria (Griffiths and Philippot, 2013). Therefore, *Proteobacteria* comprises the majority of members with a wide range of habitats. For example, the relative abundance of *Alphaproteobacteria* and its member *Sphingomonadales* significantly decreased after afforestation, but its another member *Rhizobiales* exhibited a distinct increasing trend (Figure 3). *Alphaproteobacteria* cannot be assigned into oligotrophic or copiotrophic categories (Fierer et al., 2007). Afforested soils had higher relative abundance of *Rhizobiales* compared with CL, which could be probably attributed to the increase in root biomass and exudate after afforestation and the removal of maize when harvested in CL. *Rhizobiales* can promote plant growth (Zhou and Fong, 2021) and participate in nitrogen fixation in soil. Other study also found similar result that the Rhizobiales populations increased after vegetation recovery on an abandoned land in the karst region (Li et al., 2021a). Moreover, the application of chemical fertilizers in combination with animal or human excreta in CL (Lan, 2021) enhanced the abundance of *Proteobacteria* (Yan et al., 2020). We also found that the most dominant phylum *Acidobacteria* did not change after afforestation, but the class *Acidobacteriae* and order *Bryobacterales* from *Acidobacteria* declined greatly after afforestation. This finding may be related to the abundance of *Acidobacteria* in soils with low substrate availability (Fierer et al., 2007). *Gemmatimonadota* belongs to copiotrophic groups (Zhang et al., 2021) and likely prefer nutrient-rich soils. Thus, the higher relative abundance of *Gemmatimonadota* was observed in afforestation soils compared with that in farmland in the Loess Plateau in China. However, the *Gemmatimonadota* population remarkably decreased after afforestation, although abundant nutrients (e.g., higher carbon) were found in afforested soils than in CL (Figure 2). This finding is consistent with the study conducted by Wu et al. (2019), who reported the higher relative abundance of *Gemmatimonadota* in agricultural soil than that in natural secondary forest, coniferous plantation, and shrubland. The members of *Gemmatimonadota* are aerobes, that are widely distributed in agricultural soils (Li et al., 2020; Sun et al., 2020). For example, *Gemmatimonadetes* has usually been investigated to be abundant in fertilized arable soil (He et al., 2020). Although *Bacteroidota* exhibited copiotrophic properties, its subgroup *Bacteroidia* class and *Chitinophagales* order remarkably decreased after afforestation. Fertilizer application in agricultural soil could enhance the *Bacteroidota* population compared with that in non-disturbed grassland (Acosta-Martínez et al., 2008). As such, higher abundance of *Bacteroidota* was observed in CL compared with that in afforested soils.

The relative abundance of *Chloroflexi* was the highest in MF, followed by CL and the lowest in NF (Figure 3). This finding could be attributed to different changes in the members of *Chloroflexi*; that is, *Dehalococcoidia* class and its branch *S085* order obviously increased, but Chloroflexia class and its branch *Thermomicrobiales* order, and *TK10* class remarkably decreased after afforestation (Figure 3). *Chloroflexi* belongs to oligotrophic phylum and is likely to be more abundant in nutrient-poor soils (Choudhary et al., 2021); thus, higher relative abundance of *Chloroflexi* and its subdivisions in CL and MF was found than that in NF. Moreover, most *Chloroflexi* can well survive at various nutrition levels by generating energy through solar radiation and 3-hydroxypropionate bi-cycle (Berg, 2011; Klatt et al., 2013). Hence, the lower percentage of canopy will increase the abundance of *Chloroflexi* in MF and CL than in NF because soils in MF and CL are exposed to more sunlight. This finding was confirmed by Liu et al. (2020b), who found the higher relative abundance of *Chloroflexi* in oil than in other plantations because of the lowest canopy percentage.

The relative abundance of *Latescibacteria* (1.29%–2.46%) was relatively low at the phylum level, but this taxa should not be overlooked because *Latescibacteria* and its subgroups were sensitive to afforestation (Figure 3). *Latescibacteria* tends to prefer anaerobic and nutrient-rich environments (Farag Ibrahim et al., 2017); therefore, its population was higher in afforested soils than in CL soils because afforestation increased the carbon content in this study. We also observed that the abundance of *Methylomirabilota* increased after afforestation possibly because of its positive correlation with carbon content (Figure 5). Notably, *Actinobacteriota* showed no significant change but tended to increase after afforestation (Figure 3). This finding could be attributed to different changes in the members of *Actinobacteriota*. In detail, the class *Rubrobacteria* and its branch order *Rubrobacterales* declined greatly, but class *MB-A2-108* and order Gaiellales, *norank_c__MB-A2-108* and *Microtrichales* increased remarkably after afforestation. Our results confirmed that *Actinobacteriota* cannot be assigned into oligotrophic or copiotrophic categories (Fierer et al., 2007) because every member of Actinobacteriota is distinctly oligotrophic or copiotrophic. However, a contradicting result was found; that is, the relative abundance of *Actinobacteriota* was higher in farmland than in afforested soil (Zhao et al., 2018b).

### Organic Carbon Variations Associated With Soil Bacterial Community

Soil microbes are primary drivers in ecosystem processes and mediate soil organic matter decomposition; as such, soil microbes play a key role in carbon biogeochemical cycles and dynamics in terrestrial ecosystems (Zhao et al., 2018b; Wang et al., 2019b). However, such responses may differ with changes in plant cover and composition (Yan et al., 2018; Zhao et al., 2018b). Afforestation induces changes in the soil bacterial community, which may affect the composition and content of SOC pools (Zhao et al., 2018b). In our present study, significant relationship was found between SOC, EOC, and POC content and bacterial abundance after afforestation. Considerable variations in carbon inputs from litter and root after afforestation can affect the utilization by bacterial community and finally influence SOC composition and distribution and their decomposability (Ren et al., 2018). In the present study, the abundance of *Methylomirabilota*, *Latescibacterota*, class *Methylomirabilia*, *MB-A2-108*, *norank_Latescibacterota*, and *Dehalococcoidia*, especially the order *Rokubacteriales* of *Methylomirabilota*, *Gaiellales*, *Microtrichales* and *norank_c__MB-A2-108* of *Actinobacteriota*, *Rhizobiales* of *Proteobacteria*, *norank_c__norank_p__Latescibacterota* of *Latescibacterota* and *S085* of *Chloroflexi* markedly increased after afforestation (Figure 4), thereby increasing the contents of SOC and its fractions. Therefore, these bacterial taxa may promote the increase in SOC fractions. In detail, the order *Rhizobiales*, a branch of *Alphaproteobacteria*, can promote plant growth (Zhou and Fong, 2021), and participate in nitrogen fixation; their abundance was positively correlated with the contents of carbon and nitrogen (Santoscaton et al., 2018). This order may be stimulated by plant roots and favored the soil with liable carbon. The coupled accumulation of carbon and nitrogen in soil following vegetation on cropland in karst regions was reported in our previous study (Lan et al., 2020). Therefore, *Rhizobiales* may play a crucial role in the restoration of degraded soil after afforestation in karst regions by improving SOC storage through nitrogen fixation. Moreover, *Methylomirabilota* was involved in nitrogen cycling and had a positive impact on SOC fractions. Therefore, members of this phyla may promote increases in SOC fractions. *Latescibacteria* favors the soil with anaerobic and eutrophic environments and can degrade various kinds of proteolytic substances and polysaccharides (Liu et al., 2020b). Higher abundance of *Latescibacteria* may increase the EOC. In particular, the abundance of *Actinobacteriota* showed no significant increase after afforestation (Figure 3) but had a positive effect on SOC and POC (Figure 5). This might be correlated with its composition. Three orders (*Gaiellales*, *Microtrichales*, and *norank_c__MB-A2-108*) of *Actinobacteriota* had significant effects on SOC fractions. *Chloroflexi* is involved in metabolic processes, such as degradation of fatty acids and aromatic compounds (Zhou and Fong, 2021). Therefore, *Dehalococcoidia* and *S085*, belonging to *Chloroflexi*, were positively correlated with SOC fraction. However, other remarkable decrease in the abundance of bacteria taxa, particularly for *Gemmatimonadota*, *Bacteroidota*, *Chloroflexi*, *Acidobacteriae*, *Rubrobacteria*, and their members showed negative relationship to SOC fraction, indicating that these taxa mediate the balance of SOC dynamics. Large amounts of plant residue inputs after afforestation may decrease the efficiency of carbon utilization or soil organic matter decomposition by soil microbes, ultimately resulting in reduced soil carbon storage (Fontaine et al., 2007). *Gemmatimonadaceae*, an order of *Gemmatimonadota*, had a biomineralizing action, resulting in the formation of calcium and magnesium carbonates (Xu et al., 2021), thereby decreasing the Ca^2+^ content through precipitation. The Ca^2+^ content was enriched in karst soil and can promote SOC accumulation (Yang et al., 2016; Xiao et al., 2017; Hu and Lan, 2019). *Bacteroidota* possibly had a strong contribution to the mineralization of soil organic matter (Li et al., 2020). Thus, a decrease in the abundance of *Bacteroidota* and its branch *Bacteroiaia* and *Chitinophagales* led to the negative responses of SOC fractions. *Acidobacteriae* belonging to *Acidobacteriota* can decompose cellulose and thrive to live in an environment with low organic carbon content (Qiu et al., 2019). The negative effects of decreased content of organic carbon was observed across land-use change in karst soil (Qiu et al., 2019). Altogether, afforestation-induced changes in plant inputs, thereby greatly influencing the dominant bacterial communities and the abundance of bacterial species. The functions of these species can then feedback to help sustain the carbon balance.

Afforestation induces plant cover change and greatly shifts soil bacterial community structure, thereby promoting OC storage and physiochemical protection in aggregates (Garcia-Franco et al., 2015; Zhao et al., 2018a). The markedly increased bacterial abundance after afforestation showed close relationship to OC content in aggregates (Figure 4), suggesting that the dominant soil bacterial taxa can significantly affect the aggregate-associated OC content, which could enhance organic carbon accumulation. Our result agreed with a previous study conducted by Zhao et al. (2018a), who stated that the increase in soil bacterial abundances after afforestation on the Loess Plateau could enhance carbon sequestration by affecting the OC content in aggregates. Afforestation promotes large amounts of fresh organic carbon input by dense roots and more plant litter, which are a major source of labile OC for bacterial growth and activity; OC binds to fine particles and is then incorporated into microaggregates and macroaggregates, thereby boosting the aggregation of soil particles and increasing the stability of soil aggregates (Tisdall and Oades, 1982; Six et al., 2000). In our previous study, we found that newly derived OC was mostly incorporated into the LMA, SMA, and MI fractions after afforestation at the same sties (Lan et al., 2022). Therefore, a major source of labile OC can be found in LMA, SMA, and MI fractions, which explains that some certain bacterial taxa such as *Rhizobiales* and Microtrichales had significant positive effects on OC content in all aggregates (Figure 5). However, microbial community structure is dependent on aggregate size due to the heterogeneity of niches and physicochemical properties (Lin et al., 2019). Macroaggregates are generally rich in more labile carbon (Six et al., 2004; Trivedi et al., 2015; Lin et al., 2019), while microaggregates contain more refractory carbon (Trivedi et al., 2017; Lin et al., 2019). Some bacteria showed strong relationship to LMA–OC and SMA–OC, while some were more closely with MI-OC and SC–OC in the present study. For example, stronger relationship between *Rhizobiales* and LMA-OC than that between OC content in smaller aggregates indicated that afforestation favored the growth of *Rhizobiales*, which is associated with plant growth; the effects of plant roots on microbes tend to decrease with decreasing aggregate size (Lin et al., 2019). On the contrary, *Dehalococcoidia*, *Microtrichales*, and *MB-A2-108* might be involved in recalcitrant carbon, which might explain their stronger relationship to MI–OC and SC–OC than to LMA–OC and SMA–OC. *Microtrichales* and *MB-A2-108* belonging to *Actinobacteriota* and *Dehalococcoidia* belonging to *Chloroflexi* can degrade recalcitrant substances (Zhang et al., 2016; Guo and Zhou, 2020; Zhou and Fong, 2021).

### Changes in Aggregate Stability Were Linked to Soil Microbial Community

Soil aggregate formation and stabilization are controlled by complex interactions between abiotic and biotic factors, e.g., soil organic carbon, microorganisms (activity and community), inorganic binding agents, and plants (Six et al., 2004; Cania et al., 2019; Dou et al., 2020; Xiao et al., 2020). In the present study, afforestation-induced changes in plant characteristics mainly exerted direct influence on bacterial communities (Figure 4). Variations in bacterial communities mainly exerted indirect effect on the stability of aggregates (MWD and MGD). In the present study, 4 of the 11 dominant bacterial phyla, 8 of the 21 dominant bacterial classes, and 10 of the dominant 28 bacterial orders had significant effects on MWD and MGD (Figure 5). Most of these bacteria showed remarkable changes due to afforestation. The stability of aggregates (MWD and GMD) was closely associated with soil bacterial abundance (Rahman et al., 2017; Zhao et al., 2018a). Soil microorganisms are critical factors that influence soil aggregation and aggregate stability due to their biomass, community structure, and metabolic products (Six et al., 2004; Rashid et al., 2016). According to the conceptual model of Golchin et al. (1994), fresh and labile organic matter favors microorganism growth, thereby stimulating the soil microbiota, increasing soil microbial activities, and promoting the stability of soil aggregates (Guo et al., 2020; Hu et al., 2020). On the one hand, afforestation induces increase in fresh organic material from litter input, root biomass, and exudates (Lan, 2020; Lan et al., 2022), thus promoting SOC and liable OC accumulation (Figure 2). Fresh organic matter formed hot spots of microbial activity (Rahman et al., 2017), which is a key factor that affects changes in soil aggregate stability (Duchicela et al., 2012). On the other hand, the rapid growth of soil bacteria and bacterial activity influence the production of exudates and hyphae, mostly involving polysaccharides, which in turn act as binding agent in soil aggregation (Tisdall and Oades, 1982; Rahman et al., 2017). Soil bacteria can generate exopolysaccharides and lipopolysaccharides, which stabilize soil aggregates by “gluing” soil particles together (Six et al., 2004; Cania et al., 2019). Our study found that certain indicator taxa including *Methylomirabilota* and its subgroups (*Methylomirabilia* and *Rokubacteriales*), *Latescibacterota*, and order *Rhizobiales* changed significantly after afforestation and had positive effects on MWD and MGD (Figures 3, 5). We inferred that these bacteria can synthesize exopolysaccharides and lipopolysaccharides during their growth and organic material decomposition, thereby enhancing soil aggregation and aggregate stability. However, certain indicator taxa including order *Bryobacterales*, *Gemmatimonadales*, *norank_c__TK10*, *Rubrobacterales*, *Thermomicrobiales*, class *Chloroflexia*, *Rubrobacteria*, and *Acidobacteriae* showed negative impacts on MWD and MGD. Microorganisms can directly consume organic binding agents, which hold aggregates together, leading to their decomposition (Li et al., 2018). Moreover, organic material mainly derived from microorganisms, polysaccharides formed during organic residue decomposition, and products during plant and microbial process undergo decomposition (Six et al., 2004). Soil bacteria can indirectly affect soil aggregation through SOC (Wang et al., 2019a). Soil organic matter can act as a major binding agent that promotes soil aggregate formation and is closely correlated with aggregate stability (Tisdall and Oades, 1982; Six et al., 2004; Rashid et al., 2016). Afforestation increased certain bacterial taxa, accompanied with increases in SOC and liable OC (Figure 2). Most of these bacteria significantly affect aggregate stability (Figure 5). Our previous research found that EOC showed the strongest effect on MWD (Lan, 2021), as confirmed by other studies that reported remarkable correlations between labile OC and soil aggregation (Tisdall and Oades, 1982; Bhattacharyya et al., 2012). The results suggest that soil bacteria can mediate the stability of aggregates by affecting soil organic matter.

## Conclusion

Afforestation on cropland can significantly alter the relative abundance of the dominant soil bacteria at the phylum, class, and order levels in the karst region. Such alterations had remarkable effects on the content of SOC fractions in bulk soil and OC content in aggregates as well as on soil aggregate stability (MWD, MGD), indicating the importance of soil bacteria for regulating SOC storage and soil aggregate stability. In detail, increases in the abundance of certain bacterial taxa such as *Methylomirabilota*, *Latescibacterota*, *Methylomirabilia*, *MB-A2-108*, *norank_Latescibacterota*; *Dehalococcoidia*, *Rokubacteriales*, *Gaiellales*, *Microtrichales*, *norank_c__MB-A2-108*, *Rhizobiales*, *norank_c__norank_p__Latescibacterota*, and *S085* increased the SOC fractions and aggregate-associated OC content after afforestation. In addition, increases in the relative abundance of *Methylomirabilota*, *Methylomirabilia*, *Rokubacteriales*, *Latescibacterota*, and *Rhizobiales* may enhance soil aggregate stability. Furthermore, our result suggests that the interaction between SOC fractions, OC content in soil aggregates, soil aggregate stability and soil bacterial community after afforestation might be a key factor for sustainable forest management in the karst region.

## Data Availability Statement

The datasets presented in this study can be found in online repositories. The names of the repository/repositories and accession number(s) can be found at: NCBI BioProject—PRJNA820133.

## Author Contributions

JL contributed to conceptualization, data curation, visualization, writing—original draft and review and editing, and supervision. SW contributed to data curation, visualization, and methodology. JW contributed to data curation and visualization. XQ contributed to formal analysis and visualization. QL contributed to visualization. MH contributed to investigation. All authors contributed to the article and approved the submitted version.

## Funding

This work was supported by the National Natural Science Foundation of China (No. 42177446; 41601584), Guizhou Provincial Science and Technology Foundation (Qiankehe Foundation [2017]1417).

## Conflict of Interest

The authors declare that the research was conducted in the absence of any commercial or financial relationships that could be construed as a potential conflict of interest.

## Publisher’s Note

All claims expressed in this article are solely those of the authors and do not necessarily represent those of their affiliated organizations, or those of the publisher, the editors and the reviewers. Any product that may be evaluated in this article, or claim that may be made by its manufacturer, is not guaranteed or endorsed by the publisher.

## Footnotes

^1^ http://rdp.cme.msu.edu/
